# An old drug, a classic signature

**DOI:** 10.1007/s12471-026-02064-4

**Published:** 2026-07-24

**Authors:** Francisco Rodrigues dos Santos, Mariana Duarte Almeida, Davide Moreira, João Miguel Santos, Inês Fiúza Pires

**Affiliations:** Unidade Local de Saúde Viseu Dão Lafões, Viseu, Portugal

A 75-year-old woman with paroxysmal atrial fibrillation, moderate aortic stenosis, and chronic obstructive pulmonary disease presented to the emergency department with a 2-day history of abdominal discomfort, nausea, vomiting, anorexia, and marked asthenia. She had been discharged 4 days earlier following a prolonged hospitalization for respiratory disease. Her medication, recently adjusted before hospital discharge, included inhaled triple therapy, rosuvastatin/ezetimibe, valsartan/hydrochlorothiazide, dapagliflozin, furosemide, pantoprazole, and short-acting bronchodilators as needed. She was on long-term oxygen therapy and nocturnal non-invasive ventilation.

On examination, she was somnolent but oriented, pale, and mildly dehydrated. Vital signs were stable, with an irregular heart rhythm and a systolic murmur. Laboratory evaluation revealed acute kidney injury with hyperkalaemia (6.2 mEq/L), with no evidence of systemic inflammation.

An electrocardiogram (ECG) was performed (Fig. 1; [1]).

**Fig. 1:**
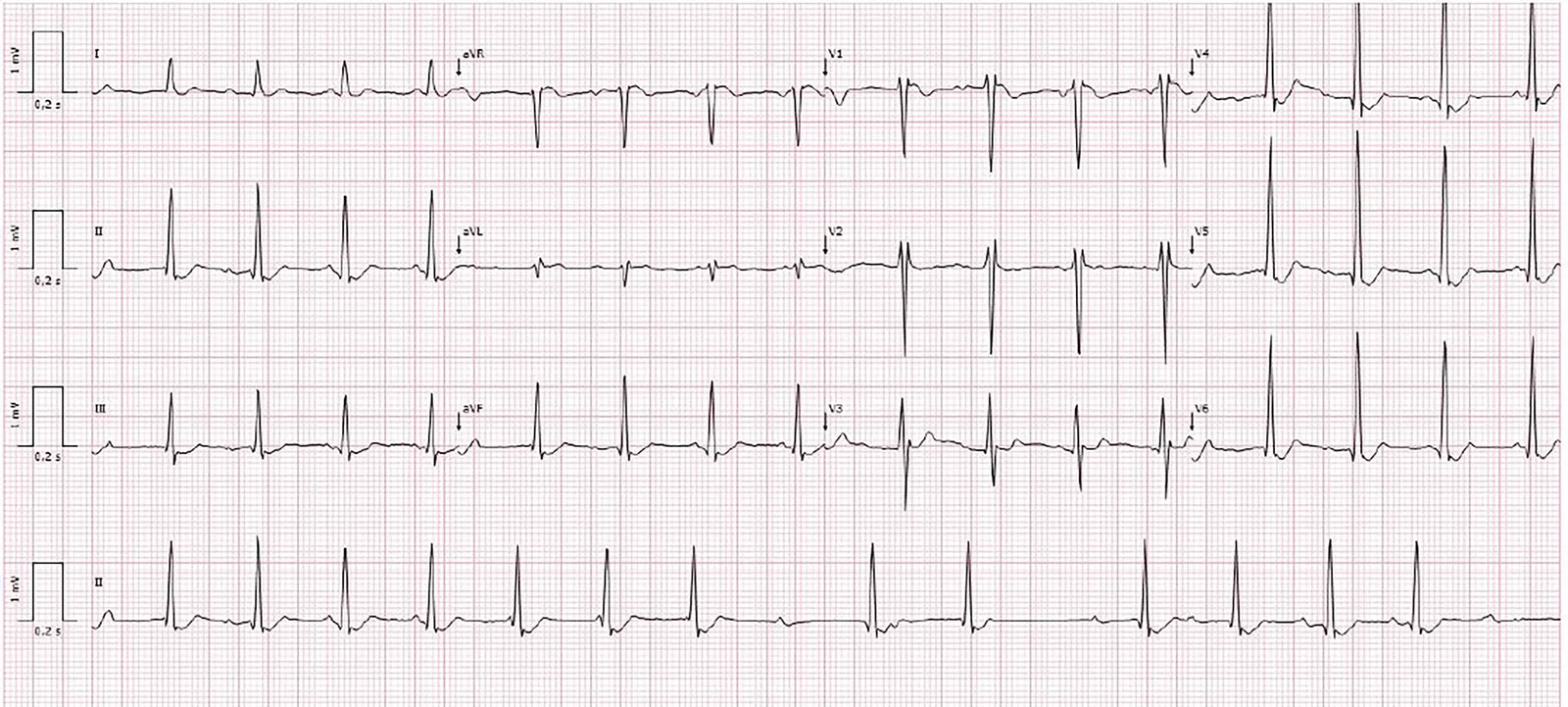
ECG at admission

*What abnormalities are present on the ECG?*

*What is the most likely diagnosis?*

## Answer

You will find the answer elsewhere in this issue.

## Data Availability

All data supporting the findings of this work are included in the article.
